# Monthly Summary

**Published:** 1871-08

**Authors:** 


### MONTHLY SUMMARY.

---

*Carbuncles.*—As carbuncles often follow each other in the same patient, anything that promises to arrest them would be gladly tried by the sufferers. Dr. Marcet suggests in the "*Lancet*" a ready method, provided it be employed as soon as the small vesicle appears on the skin.

He says :—"If the carbuncle be allowed to proceed, say, for twelve hours beyond its very first appearance, it will run its usual course ; but its progress may be arrested by the early destruction of the vesicle and its contents by means of the cauterizing action of heat. I have adopted many plans to effect this purpose ; but the simplest of all, and one which may be considered as always at hand, is the use of an incandescent lucifer-match. The vesicle is to be merely touched, for a fraction of a second, with the red-hot point from five to seven or eight times in succession, when it assumes a dull-whitish appearance from the coagulation of the album it contains. The end of the hot wire may also be used. The pain of the operation is really trifling, and it will soon save from a week to a fortnights suffering. I have repeatedly applied this form of actual cautery to myself, and shall not hesitate to do so again if necessary.

"In general, within four or five hours after the operation, the pain from the incipient carbuncle has in a great measure disappeared, and there is an end to it. It may happen, however, that the carbuncle at its origin, is deep under the surface of the skin, when no vesicle appears. I have not been so successful with the use of the actual cautery in these cases as in the others ; but probably, had the cauterization been carried deeper, the mischief might have been arrested."

Dr. Marcet has tried nitric acid, and nitrate of silver, but found them unreliable. He thinks the early vesicle may contain a virus, by destroying which the disease is nipped in the bud. This simple mode is likely to be tried further.

Dr. J. C. Nott, in the *New York Medical Journal* for January, records a case which he says is "the only real abortion of a carbuncle he ever saw." It was three inches in diameter, and involved the tissues very deeply. He made a deep incision of one and a quarter inches, and stuffed it with cotton saturated with pure carbolic acid, and also painted the whole hardened surface with the remedy. Dr. Nott says :—The patient complained of a sharp burning sensation for a few minutes, when the pain subsided completely. The cuticle, by the next day, came off, and the surface looked like a burn. After the first few minutes he was free from pain, and never complained of any afterward. I continued every day for a week to insert the acid, in the same way, into the cut, which sloughed all around to the depth of one-eight of an inch ; the surrounding inflammation and induration subsided rapidly, and in a week there was nothing left to treat, but the small open wound made by the knife and acid. Three other small carbuncles commenced, an inch or two from the large one; they were all treated by incision and the acid, and they all aborted."—*The Doctor.*

---

*Concealed Vascular Tumor of the Face.*—Dr M. Townsend reported at a clinic of Prof. Gross, Jefferson Medical College, as published in the *Medical Times*, the case of a patient, aged 54 who had had a tumor on his face for upwards of a year, supposed to be sebaceous, having the feel and external characteristics of a growth of this kind. On cutting into it, however, it proved to be a vascular tumor, and some little time was occupied in controlling the resulting hæmorrhage. Needles, armed with strong ligatures, were passed crucially under the mesh of arteries and veins, and the growth was then, subcutaneously as it were, thoroughly ligated.

Such affections as these are generally congenital. These tumors exhibit considerable variety of structure, being sometimes essentially composed of veins, sometimes nearly equally of arteries and veins. When the tumor is arterial, it generally pulsates synchronously with the left ventricle of the heart.

---

*Condurango.*—Some of our exchanges (*Nat. Med. Jour.*, *Medical Gazette*, *Boston Medical and Surgical Journal*) refer to a recent correspondence between the Minister of the United States to the Republic of Ecquador and the government at Washington in regard to the introduction into this country for medicinal purposes of the wood of a tree called Condurango. It is said that fifty pounds of the wood have been placed at the disposal of the Surgeon-General of the navy for experiment by naval surgeons and other medical men, and it is the expressed wish of the Secretary of State that Mr. Robeson should cause it to be administered by the medical staff of the navy. The diseases in which the drug has been found to act most happily by the physicians of Ecquador are syphilis and cancer,—two diseases not generally thought to be closely allied. One of our cotemporaries says:

"Unfortunately, the accounts of cures affected by this drug have been derived in great part from ignorant people, and the word cancer is so loosely used that it is impossible to believe in all the cases in which the patient was so fortunately restored to health under its use, that he was affected with malignant disease."—*Med. Times.*

---

### EDITORIAL DEPARTMENT.

